# Natural killer cells at the interface of tumor immune escape and immunotherapy resistance in endometrial cancer

**DOI:** 10.3389/fimmu.2026.1784443

**Published:** 2026-08-07

**Authors:** Alessandro Poggi, Valentina Bruno, Anna Di Spirito, Lorenzo Mortara, Denisa Baci

**Affiliations:** 1 Molecular Oncology and Angiogenesis Unit, IRCCS Ospedale Policlinico San Martino, Genoa, Italy; 2 Gynecologic Oncology Unit, Department of Experimental Clinical Oncology, IRCCS Regina Elena National Cancer Institute, Rome, Italy; 3 Immunology and General Pathology Laboratory, Department of Biotechnology and Life Sciences, University of Insubria, Varese, Italy; 4 Molecular Cardiology Laboratory, IRCCS Policlinico San Donato, Milan, Italy

**Keywords:** endometrial cancer, immune escape, immunotherapy, natural killer cells, tumor microenvironment

## Abstract

Endometrial cancer (EC) is characterized by significant molecular and immunological heterogeneity, which influences both disease progression and response to therapy. Although immune checkpoint blockade has improved the clinical management of selected EC subtypes, durable responses remain limited in many patients, particularly in tumors with poorly inflamed or mismatch repair-proficient/microsatellite-stable profiles. In this context, natural killer (NK) cells represent an important but still insufficiently explored component of anti-tumor immunity. NK cells can recognize transformed or stressed cells independently of antigen-specific priming, which may be relevant in EC tumors characterized by altered antigen presentation, immune exclusion, or limited T-cell responsiveness. Within the EC tumor microenvironment, NK-cell dysfunction is likely shaped by a complex interplay between defective recruitment, altered receptor–ligand interactions, suppressive cytokine networks, and tumor-driven immune remodeling. These mechanisms may reduce NK-cell cytotoxicity and favor immunoregulatory or tolerance-like phenotypes, some of which resemble programs involved in maternal–fetal immune tolerance. Here, we examine how EC may reshape NK-cell recruitment, phenotype, and function, and discuss how these alterations intersect with molecular tumor heterogeneity, immune escape, and emerging NK-directed therapeutic strategies. Particular attention is given to how NK-directed and NK-complementary approaches, including cytokine-based activation, NK-cell engagers, adoptive NK-cell transfer, chimeric antigen receptor natural killer cell (CAR-NK) platforms, and their integration with immune checkpoint blockade, may help address resistance in EC.

## 1 Introduction

Endometrial cancer (EC) is the sixth most commonly diagnosed cancer in women and represents one of the most rapidly increasing gynecological malignancies worldwide (1). Its rising incidence is largely attributed to increasing life expectancy and lifestyle-related risk factors, including obesity, metabolic syndrome, and hormonal imbalance, which affect both postmenopausal and, increasingly, younger women (2). Age remains an adverse prognostic factor, as aging is frequently associated with comorbidities, metabolic dysfunction, reduced overall survival (OS), and more aggressive tumor features.

EC is often diagnosed at an early stage because of abnormal uterine bleeding, allowing favorable clinical outcomes in many patients. However, advanced, recurrent, or metastatic EC remains associated with poor prognosis and limited therapeutic options. Patient outcome and treatment selection are strongly influenced by clinical parameters, histological subtype, and molecular features. The 2021 European Society of Gynaecological Oncology/European Society for Radiotherapy and Oncology/European Society of Pathology (ESGO/ESTRO/ESP) guidelines incorporated pathological and molecular parameters into EC management (3). These recommendations were further refined by the 2023 International Federation of Gynecology and Obstetrics (FIGO) staging system, which integrates molecular classification, histological subtype, lymphovascular space invasion, and updated anatomical descriptors, including peritoneal spread and nodal disease (4, 5). Together, these advances aim to improve risk stratification, guide treatment personalization, and optimize clinical outcomes (6).

The standard management of EC includes surgery, radiotherapy, and chemotherapy (7). Surgery remains the mainstay of treatment for early-stage disease, with minimally invasive approaches increasingly used in clinical practice (8). Radiotherapy and chemotherapy improve disease control in selected settings, but their efficacy is limited by toxicity, recurrence, and drug resistance (9, 10).

More recently, targeted therapies and immunotherapies have reshaped the treatment landscape of advanced EC. In particular, immune checkpoint inhibitors (ICIs) targeting the programmed cell death protein 1/programmed death-ligand 1 (PD-1/PD-L1) pathway have shown clinically relevant activity, especially in mismatch repair-deficient/microsatellite instability-high (dMMR/MSI-H) tumors (11, 12). Combination strategies, such as pembrolizumab plus lenvatinib, have expanded therapeutic options for advanced disease, including patients with mismatch repair-proficient/microsatellite-stable (pMMR/MSS) disease (11). Nevertheless, a substantial proportion of patients remains refractory or develops resistance, highlighting the need for additional immune-based approaches tailored to the tumor microenvironment (TME).

EC arises from a complex interaction between tumor cells and the immune system, in which immune surveillance is progressively counteracted by immune evasion mechanisms (13). Although most immunological studies in EC have focused on adaptive immunity, particularly T lymphocytes, innate immune cells also contribute to tumor progression, immune escape, and therapy response. Among them, natural killer (NK) cells are of particular interest because of their ability to recognize and eliminate transformed cells without prior antigen sensitization.

NK cells are cytotoxic innate lymphocytes that integrate activating and inhibitory signals to control tumor cell recognition and killing. In the healthy endometrium, uterine NK cells contribute to tissue remodeling, vascular regulation, immune homeostasis, and pregnancy-associated decidualization. These physiological functions are closely linked to maternal–fetal tolerance, a process that EC may partially exploit to suppress anti-tumor immunity (13, 14). Within the EC TME, NK cells may undergo altered recruitment, phenotypic remodeling, impaired cytotoxicity, and functional polarization toward poorly cytotoxic or pro-angiogenic states. Understanding how EC reshapes NK-cell biology is therefore essential to define new strategies capable of restoring NK-cell-mediated anti-tumor activity.

This review focuses on the biological role of NK cells in the endometrium and in EC, with particular emphasis on how the EC TME affects NK-cell recruitment, phenotype, and function. We discuss the relationship between NK-cell dysfunction, immune escape, and the molecular heterogeneity of EC. Current and emerging NK-targeted therapeutic strategies are also reviewed, including cytokine-based activation, NK-directed checkpoint blockade, NK-cell engagers, adoptive NK-cell transfer, and chimeric antigen receptor natural killer cell (CAR-NK) platforms, positioning NK-cell-directed strategies within the evolving immunotherapy landscape.

## 2 Molecular landscape and biomarkers in endometrial cancer

EC is a heterogeneous disease characterized by distinct molecular alterations that influence tumor behavior, prognosis, immune phenotype, and therapeutic response. The traditional Bokhman classification has largely been replaced by molecular taxonomy. In 2013, The Cancer Genome Atlas (TCGA) defined four major EC subtypes: POLE-ultramutated, microsatellite instability-high/mismatch repair-deficient (MSI-H/dMMR), copy-number low/no specific molecular profile (NSMP), and copy-number high/p53-abnormal tumors (15, 16). This classification was later adapted into clinically applicable systems, including ProMisE, and is now incorporated into diagnostic and therapeutic guidelines together with histological and clinicopathological parameters (17–19).

These molecular subtypes differ in both clinical behavior and immune contexture. POLE-ultramutated tumors are characterized by pathogenic mutations in the exonuclease domain of DNA polymerase epsilon, resulting in very high tumor mutational burden (TMB), abundant neoantigen formation, and excellent prognosis (5, 16, 20). They are generally considered immune-hot tumors, with prominent CD4^+^ and CD8^+^ tumor-infiltrating lymphocytes and increased expression of immune checkpoint molecules such as PD-1 and PD-L1 (21, 22). MSI-H/dMMR tumors are also highly immunogenic because of defective DNA mismatch repair, frequent hypermutation, and increased checkpoint expression (23–25). This subgroup is clinically relevant because dMMR/MSI-H status predicts response to ICIs, including pembrolizumab and dostarlimab (26, 27).

By contrast, NSMP tumors are molecularly heterogeneous and frequently harbor alterations in PTEN, PIK3CA, KRAS, ARID1A, or CTNNB1, reflecting activation of PI3K/AKT/mechanistic target of rapamycin (mTOR) and Wnt/β-catenin pathways (18). Their prognosis is intermediate but variable, with CTNNB1 mutations associated with increased recurrence risk (28, 29). The p53-abnormal subtype is characterized by TP53 alterations, chromosomal instability, aggressive clinical behavior, and poor prognosis (15, 16). HER2 amplification may occur in a subset of p53-abnormal/serous ECs, supporting HER2-targeted treatment in selected patients (30).

Several biomarkers currently guide prognosis and therapeutic decisions in EC, including MMR status, POLE mutations, TP53 alterations, PD-L1 expression, TMB, and ARID1A loss (31–35). Among these, dMMR/MSI-H status remains the most established predictive biomarker for response to ICIs in clinical practice (31, 32). Current immune stratification is still largely based on T-cell infiltration, TMB, MMR status, and checkpoint expression: POLE-mutated and MSI-H/dMMR tumors are generally considered immune-hot, whereas many NSMP and p53-abnormal tumors display less inflamed, immune-excluded, or immunosuppressive features (36, 37). This distinction has therapeutic relevance, as pembrolizumab plus lenvatinib has shown activity in advanced pMMR EC, likely through modulation of the TME (11).

However, NK-cell-related parameters remain poorly integrated into this molecular and immune framework. Although subtype-stratified studies have defined major differences in global immune contexture, the distribution of NK-cell abundance, functional state, activating and inhibitory receptor expression, and NK-relevant ligand profiles across EC molecular subtypes remains insufficiently characterized. Future subtype-annotated studies should therefore incorporate NK-cell phenotyping, receptor–ligand profiling, and spatial immune analyses to determine whether specific EC subgroups may benefit from NK-directed or NK-complementary immunotherapeutic strategies.

## 3 NK cells in the endometrial cancer tumor microenvironment

### 3.1 Immune infiltrates in the EC TME

**Figure 1:**
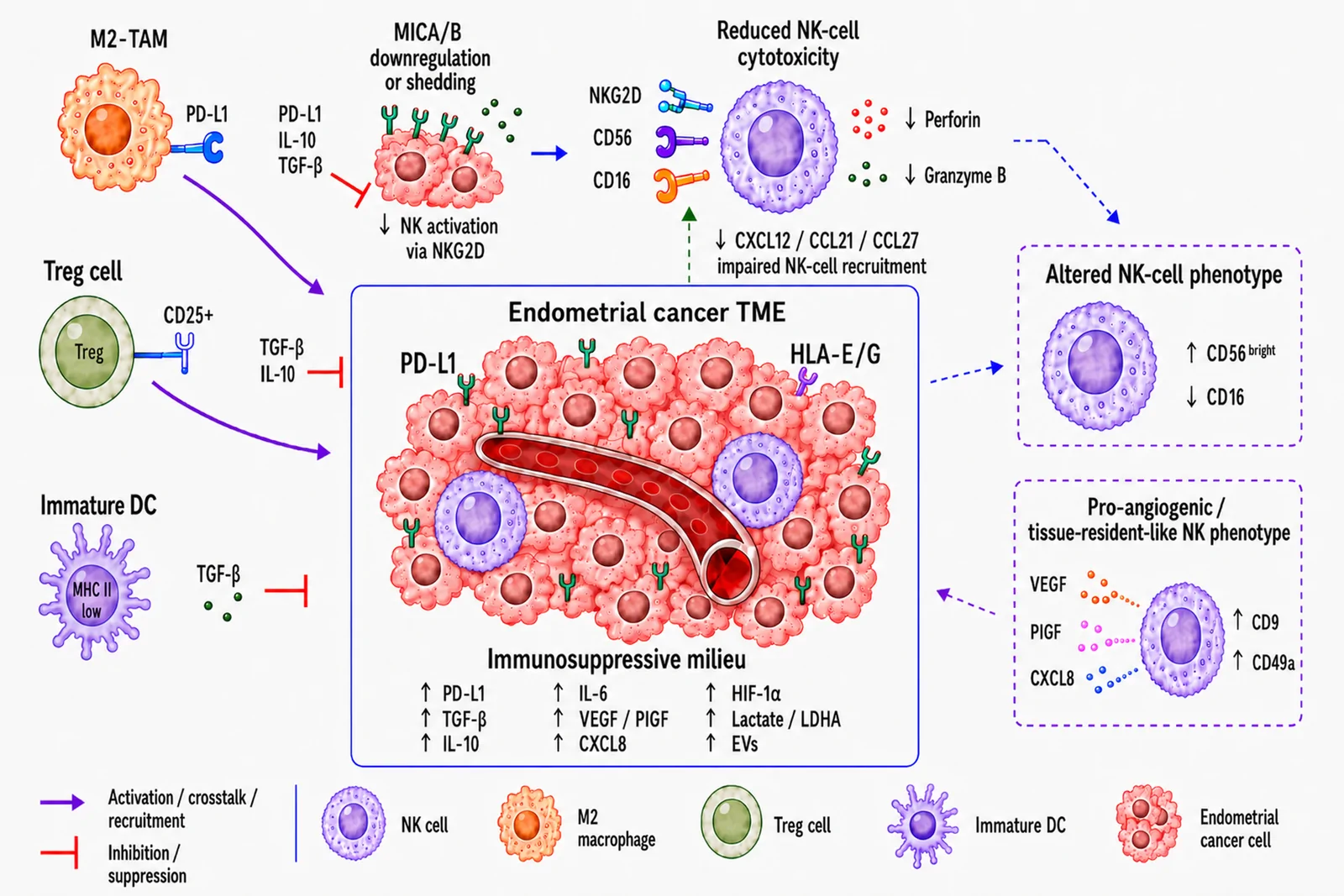
Established and proposed NK-cell immune-evasion mechanisms in endometrial cancer. The figure summarizes NK-cell dysfunction within the EC tumor microenvironment. The central panel shows EC-supported alterations, including reduced NK-cell infiltration, impaired recruitment associated with decreased CXCL12/CCL21/CCL27, reduced cytotoxic function, increased inhibitory receptor expression, and an immunosuppressive milieu characterized by TGF-β, IL-6, PD-L1, IL-10, VEGF/PlGF, CXCL8, HLA-E/G, hypoxia-related signals, lactate/LDHA, and extracellular vesicles (EVs). M2-like macrophages, Treg cells, and immature dendritic cells contribute to NK-cell suppression through inhibitory cytokines, checkpoint-ligand expression, and defective immune priming. The MICA/B–NKG2D axis is represented as a candidate NK-evasion pathway through which ligand downregulation or shedding may impair NKG2D-dependent NK-cell activation and cytotoxicity; its direct contribution in EC remains to be fully validated. Dashed phenotype panels indicate proposed NK-cell states requiring EC-specific confirmation, including an altered CD56^bright^/CD16^low^ phenotype and a pro-angiogenic/tissue-resident-like NK-cell state extrapolated from decidual NK-cell biology and other solid tumors.

The EC TME contains a diverse array of immune infiltrates, including T lymphocytes, B cells, tumor-associated macrophages (TAMs), dendritic cells (DCs), and, less abundantly, NK cells. Recent single-cell and bulk profiling studies confirm that immune infiltration in EC is heterogeneous and often skewed toward immunosuppression (38). Among these populations, TAMs frequently dominate the myeloid compartment and are often polarized toward an M2-like immunosuppressive phenotype (39) (**Figure 1**).

Recent data also link TAM polarization to suppressed nucleotide-binding oligomerization domain-like receptor protein 3 (NLRP3) inflammasome activity, suggesting that inflammasome regulation may contribute to macrophage-mediated immunosuppression in EC (40). DCs in EC may display impaired maturation or tolerogenic phenotypes, limiting effective antigen presentation and T-cell priming (41). T and B lymphocytes exert both effector and regulatory functions; for example, high densities of CD8^+^ T cells in EC correlate with a more favorable prognosis (42).

Although NK cells are modestly represented in many EC specimens, they are of particular interest due to their innate cytotoxic potential. Degos et al. demonstrated that NK-cell proportions are lower in tumor tissue than in adjacent non-tumor endometrium, suggesting either physical exclusion or selective suppression within the EC TME (43). Single-cell transcriptomic profiling has further refined NK-cell heterogeneity in EC, identifying subsets with stress/inflammatory signatures and reduced cytotoxic potential (44). Thus, the low abundance of NK cells in EC, together with their dysfunctional transcriptional state, highlights their limited but potentially critical role in the local immune milieu. Given this complex immune composition, NK cells emerge as key modulators of the EC immune contexture.

### 3.2 NK-cell biology and uterine NK-cell subsets

NK cells are innate immune cells belonging to the innate lymphoid cell (ILC) family (45, 46). They play a central role in immunosurveillance by recognizing and eliminating transformed, virus-infected, or stressed cells without prior antigen sensitization. NK-cell activity is regulated by the balance between activating receptors, including natural killer group 2D (NKG2D), CD16, and natural cytotoxicity receptors (NCRs), and inhibitory receptors (47–49). In solid tumors, NK cells mediate direct cytotoxicity and produce cytokines such as interferon gamma (IFN-γ), thereby supporting a Th1-skewed immune milieu, promoting DC and T-cell responses, and contributing to metastatic control (50–54). In EC, although direct functional evidence remains limited, NK cells may contribute to early tumor immunosurveillance before full activation of adaptive immunity.

In peripheral blood, human NK cells are classically divided into two main subsets: CD56^dim^CD16^+^ NK cells, which represent approximately 90%–95% of circulating NK cells, and CD56^bright^CD16^−/low^ NK cells, which account for the remaining 5%–10% (46, 47). CD56^dim^CD16^+^ NK cells are highly cytotoxic and can eliminate tumor cells through NKG2D, CD16, and NCR-mediated activation, as well as through the release of perforin and granzymes or death receptor–ligand interactions involving Fas ligand (FASL) and TNF-related apoptosis-inducing ligand (TRAIL) (47). Although this subset has limited cytokine-producing capacity at baseline, it can rapidly produce IFN-γ upon activation (55). In contrast, CD56^bright^CD16^−/low^ NK cells are poorly cytotoxic but are potent producers of cytokines, including IFN-γ, tumor necrosis factor alpha (TNF-α), and granulocyte–macrophage colony-stimulating factor (GM-CSF).

Similar to T cells, terminally differentiated NK cells can acquire PD-1 expression and become susceptible to inhibition through interaction with PD-L1 expressed by tumor cells (48). Indeed, the PD-1/PD-L1 axis can inactivate both T cells and NK cells, and PD-L1 expression in tumors is increasingly considered relevant for identifying patients who may benefit from monoclonal antibodies disrupting PD-1/PD-L1 interactions (48). More broadly, NK-cell activity depends on the integration of signals from activating receptors, such as NKp46/NCR1, NKp44/NCR2, NKp30/NCR3, NKG2D, and DNAX accessory molecule-1 (DNAM-1), and inhibitory receptors, including CD94/NKG2A and killer-cell immunoglobulin-like receptors (KIRs).

Single-cell transcriptomic and multiparametric phenotyping studies have refined the classification of human NK cells beyond the conventional CD56^dim^ and CD56^bright^ subsets. Rebuffet et al. identified three major NK-cell states, NK1, NK2, and NK3 (56). Although this classification is biologically informative, its relevance to EC remains uncertain since these subsets have not yet been functionally validated as an EC-specific classification in tumor tissues.

Another relevant NK-cell population is represented by endometrial/uterine NK (eNK) cells and decidual NK (dNK) cells, which display a tissue-specific receptor phenotype. During pregnancy, these cells are essential mediators of decidual vascularization and spiral artery remodeling (57, 58). Both eNK and dNK cells represent major immune populations in the human uterine mucosa in non-pregnant and pregnant women, respectively. Although several observations suggest that eNK cells may act as precursors of dNK cells, their phenotype and function remain incompletely defined. With an increasing number of pregnancies, eNK cells show higher expression of NKG2C and leukocyte immunoglobulin-like receptor B1 (LILRB1/CD85j), suggesting a trained-memory process in uterine NK cells (59). Dysregulation of the cytotoxic and regulatory NK-cell balance has also been implicated in recurrent miscarriage and preeclampsia (60).

This uterine NK-cell compartment is strongly influenced by inhibitory receptor expression, particularly KIR expression, which is shaped by maternal human leukocyte antigen C (HLA-C) and biased toward KIR2D expression (61). dNK cells can represent up to 50% of decidual lymphocytes during the first trimester of pregnancy and are characterized by a CD56^superbright^CD16^−^CD49a^+^CD9^+^ phenotype (62). dNK cells have low cytotoxic activity and secrete pro-angiogenic and immunoregulatory factors, including vascular endothelial growth factor (VEGF), placental growth factor (PlGF), interleukin-8 (IL-8)/CXCL8, angiogenin, and IL-10. These mediators are critical for decidual vascularization, spiral artery remodeling, embryo implantation, and establishment of the tolerogenic environment required to support fetal development (63–66).

Evidence that NK cells may acquire pro-angiogenic and decidual-like features in human tumors was first reported in NSCLC (67). Patients with NSCLC showed an expansion of polarized blood dNK-like cells expressing high levels of CD56, CD9, CD49a, and CXCR3, with *in vitro* pro-angiogenic capabilities resembling dNK cells (67). Hanna et al. demonstrated that co-injection of human dNK cells with JEG-3 trophoblast choriocarcinoma cells into nude mice promoted tumor growth and vascularization compared with co-injection of peripheral-blood NK cells (57). This effect was partially inhibited by co-injection of Flt1-Fc, which neutralizes VEGF and PlGF (57). Other tumor contexts were shown to contain a predominant CD56^bright^CD16^−/low^ NK-cell response with tumor-nurturing capacity, particularly through promotion of angiogenesis (65). Du et al. described a feeder-free *in vitro* system able to induce human pro-angiogenic NK cells from umbilical cord blood hematopoietic stem cells, bone marrow hematopoietic stem cells, and peripheral blood NK cells (68). These induced NK cells were predominantly CD49a^+^, displayed low cytotoxic activity, expressed high levels of CD9, CD39, and CD151, and showed low CD16 expression (68). They also released growth-promoting factors and showed upregulation of genes related to blood vessel formation and embryonic development, including VEGF, leukemia inhibitory factor (LIF), and IL-32. *In vivo* adoptive transfer experiments in a mouse pregnancy model further confirmed their pro-angiogenic function (68), supporting the relevance of CD49a in the acquisition of pro-angiogenic NK-cell activity.

We and others have demonstrated that tumor-infiltrating and peripheral NK cells may be enriched in CD56^bright^CD16^−/low^ cells with impaired activating receptor expression, reduced cytotoxic capacity, and pro-angiogenic features resembling dNK cells. These cells have been referred to as decidual-like NK cells (67, 69–78).

### 3.3 NK-cell dysfunction and immune evasion mechanisms

NK-cell function within the TME is shaped by multiple cellular and soluble components, including TAMs, DCs, myeloid-derived suppressor cells (MDSCs), endothelial cells, cancer-associated fibroblasts, T and B lymphocytes, cytokines, chemokines, and growth factors (79–81). These interactions can impair NK-cell recruitment, activation, cytotoxicity, and cytokine production (**Figure 1**).

The immunosuppressive cytokine milieu is a major driver of NK-cell dysfunction. Transforming growth factor beta (TGF-β) is one of the strongest suppressors of NK-cell activity, reducing activating receptor expression, impairing cytotoxic granule release, reprogramming metabolism, and contributing to dNK-like polarization (65, 67, 72). In EC, elevated TGF-β and IL-6 are commonly observed and may contribute to NK-cell suppression (82). Regulatory T cells (Tregs) and MDSCs may further antagonize NK cells through contact-dependent or soluble inhibitory pathways (41). Tregs may compete for IL-2 or release TGF-β and IL-10, whereas MDSCs may produce arginase, reactive oxygen species, or PD-L1, creating a hostile environment for NK-cell activation.

Tumor-associated NK cells may also acquire decidual-like and pro-angiogenic features. In different solid tumors, including NSCLC, colorectal cancer, prostate cancer, and malignant pleural effusions, NK cells with a CD56^bright^CD16^−/low^CD49a^+^CD9^+^ phenotype have been described, together with reduced cytotoxicity, impaired degranulation, and pro-angiogenic activity (67, 70, 71, 76). These cells can produce angiogenic mediators such as VEGF, PlGF, IL-8/CXCL8, angiogenin, MMP9, and tissue inhibitor of metalloproteinase-2 (TIMP-2), and may support endothelial cell migration and capillary-like structure formation (67, 70, 71). TME-related factors, including TGF-β, hypoxia, and glycodelin-A, can promote this shift toward CD56^bright^CD16^−/low^ decidual-like NK cells with pro-angiogenic functions (83–91) (**Figure 1**).

Beyond soluble cytokines and checkpoint ligands, metabolic mediators are increasingly recognized as central regulators of NK-cell dysfunction in EC and other solid tumors. Lactate is enriched in several solid TMEs and promotes immunosuppressive polarization of DCs while impairing cytotoxic T-cell and NK-cell effector functions, supporting the view that lactate is not merely an end product of glycolysis or fermentation but an active signaling metabolite that reinforces tumor fitness and weakens cell-based immunotherapies (92). High lactic acid levels can indirectly restrain NK-cell activity through expansion of MDSCs (93), and recent EC-specific evidence indicates that tumor-derived lactate drives M2-type macrophage polarization and enhances tumor progression (94).

Extracellular vesicles (EVs) are a relevant component of the EC microenvironment. EVs shuttle proteins, lipids, and nucleic acids between cells and are being investigated as both biomarkers and therapeutic tools in EC (95). In EC, EV cargo, particularly miRNAs, appears to have diagnostic and translational relevance (95, 96). Accordingly, tumor-derived EVs can remodel the TME by promoting epithelial–mesenchymal transition (EMT), angiogenesis, invasion, metastasis, and drug resistance (97). Their relationship with NK-cell biology remains complex, as EVs have been reported both to suppress and, in selected settings, to stimulate NK-cell activity (98). In EC, the biological and clinical characterization of circulating EVs is still limited compared with other solid tumors (99); however, available evidence suggests that EV-mediated communication between EC cells, cancer-associated fibroblasts, and TAMs contributes to disease progression (95, 100).

Hypoxia is another hallmark of the TME and is tightly linked to tumor angiogenesis (101). In EC, increased hypoxia-inducible factor 1-alpha (HIF-1α) activity has been associated with tumor development and progression, and high HIF-1α expression correlates with adverse clinicopathological features and poorer survival (102, 103). Hypoxia also has direct relevance for NK-cell biology: hypoxic TME conditions can reduce activating receptor expression, impair cytokine production, alter metabolism, and contribute to the emergence of poorly cytotoxic, decidual-like/pro-angiogenic NK-cell states (104).

EC-specific evidence supports altered NK-cell recruitment and function. Degos et al. showed that NK cells are reduced in EC tissue and display impaired cytotoxic function, together with altered recruitment within the TME (43). Tumor-resident CD103^+^ NK cells expressed higher levels of T-cell immunoreceptor with immunoglobulin and ITIM domain (TIGIT) and T-cell immunoglobulin and mucin domain-containing protein 3 (TIM-3) than tumor-recruited CD103^−^ NK cells, and expression of these inhibitory molecules increased with disease severity (43). The same study found increased IL-1β and IL-6 in tumor tissue compared with adjacent healthy tissue; IL-6 is a negative regulator of NK-cell function, whereas IL-1β may influence both cytotoxic responses and tumor vessel activation (43). Moreover, chemokines involved in NK-cell recruitment, including CXCL12, CCL21, and CCL27, were significantly reduced in tumors (43). These findings suggest that the EC TME reshapes NK-cell recruitment, phenotype, and cytotoxic activity, thereby favoring immune escape.

The intrinsic plasticity of uterine NK cells may further contribute to this process. A recent comparison of eNK cells from non-pregnant endometrium and dNK cells from early pregnancy showed that dNK cells upregulate activating receptors, including NKG2D, NKp30, and NKp46, and display a lower frequency of some KIR subtypes (64). Although EC-specific data remain limited, the capacity of the endometrium to generate decidual NK-cell programs suggests that EC may hijack related regulatory pathways. If EC-infiltrating NK cells acquire features resembling dNK or CD56^bright^CD16^−/low^ subsets, they may show reduced cytotoxicity, increased immunoregulatory activity, and potential pro-angiogenic functions. Thus, the CD56^bright^/CD56^dim^ balance and the functional state of NK-cell subsets may be relevant for EC progression, prognosis, and NK-based therapeutic design.

In EC and other solid tumors, non-classical HLA molecules represent another important immune-evasion mechanism. Tumor cells may express HLA-E and HLA-G, which can engage inhibitory receptors on NK cells and suppress their activation. HLA-E binds CD94/NKG2A and can inhibit NK-cell cytotoxicity (47, 48). In EC, Versluis et al. found that the prognostic impact of NK-cell infiltration was modulated by HLA-E expression: NK-cell presence was associated with improved survival when HLA-E was upregulated, whereas under normal HLA-E expression, NK infiltration was paradoxically associated with worse prognosis (105). Thus, NK-cell abundance alone is insufficient to define effective anti-tumor immunity and should be interpreted together with inhibitory ligand expression, NK-cell subset composition, and functional state.

The biological effect of HLA-E may be context dependent, as this molecule can influence both NK-cell and CD8^+^ T-cell responses (106, 107). The HLA-E/NKG2 axis is further shaped by the ability of HLA-E to bind both inhibitory NKG2A and activating NKG2C receptors, with higher affinity for NKG2A under physiological conditions (108–114). Therefore, the balance between HLA-E expression levels, receptor saturation, NKG2A/NKG2C distribution, and the local immune context may determine whether HLA-E predominantly inhibits or supports NK-cell activity (115). Further EC-specific studies are required to clarify how this axis shapes NK-cell function during tumor progression.

Another key evasion mechanism involves dysregulation of NKG2D ligands, particularly MHC class I chain-related A/B (MICA/B). Tumor cells can downregulate or shed MICA/B, generating soluble ligands that saturate or downmodulate NKG2D on NK cells and weaken activating receptor–ligand interactions (116). Inhibition of MICA/B shedding has been shown in experimental models to restore NK-cell-mediated anti-tumor immunity (116) (**Figure 1**).

Overall, NK cells in the EC TME are exposed to multiple suppressive pressures, including inhibitory checkpoint engagement, altered chemokine-mediated recruitment, suppressive cytokines, decidual-like polarization, and loss or shedding of activating ligands. These mechanisms weaken NK-cell cytotoxicity, reduce cytokine output, and limit their contribution to anti-tumor immunity. Understanding these pathways is essential for interpreting the clinical significance of NK cells in EC and for developing NK-directed therapeutic strategies.

Direct EC evidence currently supports altered NK-cell recruitment, reduced intratumoral NK abundance, upregulation of inhibitory receptors in resident NK cells, low-cytotoxic transcriptional states, and clinically relevant associations involving HLA-E/HLA-G. By contrast, TGF-β-driven decidual-like polarization, pro-angiogenic NK-cell conversion, and other tolerance-like programs remain biologically plausible but are still supported mainly by uterine physiology and cross-tumor literature rather than by definitive EC-specific spatial and functional validation.

### 3.4 Clinical relevance and evidence gaps

The clinical significance of NK-cell infiltration and function in EC is less well defined than that of T cells. However, available evidence suggests that NK cells may have relevant prognostic and therapeutic implications. Versluis et al. reported that NKp46^+^ NK-cell infiltration was associated with improved disease-free and disease-specific survival, but only in tumors with increased HLA-E expression (105). This context-dependent association indicates that NK-cell abundance alone is insufficient to define effective anti-tumor immunity and should be interpreted together with the inhibitory ligand landscape, NK-cell subset composition, and functional state.

A possible explanation for this apparently counterintuitive finding is that HLA-E expression may not act solely as a static inhibitory marker. HLA-E can engage inhibitory CD94/NKG2A receptors, but its functional effect may depend on ligand density, peptide repertoire, and the relative distribution of NKG2A- and NKG2C-expressing NK cells. Therefore, in some EC contexts, high HLA-E expression may reflect an immune-inflamed TME in which NK cells are present and biologically engaged, whereas normal HLA-E expression may coexist with poorly functional or non-cytotoxic NK-cell states. Thus, the prognostic impact of NK infiltration in EC likely depends on the combined spatial and functional organization of NK cells, HLA-E/HLA-G expression, and other inhibitory pathways rather than on NK-cell density alone (105–115).

To clarify the level of evidence supporting NK-cell involvement in EC, current findings can be grouped into four categories. First, EC patient tissue and clinical association evidence supports reduced NK-cell infiltration, altered NK-cell phenotype, and prognostic interactions with HLA-E expression (43, 44, 105, 117, 118). Tumor-resident CD103^+^ NK cells express higher levels of TIGIT and TIM-3 than recruited CD103^−^ NK cells, together with reduced cytotoxic effector production and degranulation (43). Single-cell analyses further identify transcriptionally distinct NK-cell subsets with low cytotoxic potential (44, 117). Additional EC-specific evidence implicates HLA-G and IDO as immune-regulatory molecules associated with aggressive clinicopathological features and poorer outcome (119, 120).

Second, EC functional evidence remains more limited but supports the concept that the EC TME can directly suppress NK-cell activity. EC-derived stromal or cancer-associated fibroblast components may impair NK-cell cytotoxicity through contact-dependent mechanisms, including downregulation of PVR/CD155 (121). Conversely, selected experimental approaches, including IL-27 treatment or combined rapamycin and cisplatin, have been reported to enhance NK-cell cytotoxicity against EC cells and improve anti-tumor effects in preclinical models (122).

Third, several NK-cell evasion mechanisms are currently supported mainly by extrapolation from other solid tumors. These include suppression through HLA-E–NKG2A and HLA-G–immunoglobulin-like transcript (ILT)/KIR pathways, TGF-β-driven NK-cell dysfunction, and NKG2D-ligand shedding (83–91, 116, 123). Although these pathways are biologically plausible and therapeutically relevant, their contribution requires direct validation in EC-specific models.

Fourth, the possibility that EC recapitulates maternal–fetal tolerance programs, thereby promoting decidual-like and potentially pro-angiogenic NK-cell states, should be considered a mechanistic hypothesis. This hypothesis is supported by the biology of uterine NK cells and by evidence from other tumor types, but it still requires direct spatial, phenotypic, and functional validation in EC tissues and models (57–78).

Overall, current evidence supports NK cells as relevant contributors to EC immune escape and as promising candidates for biomarker development and therapeutic targeting. Priority gaps include prospective spatial profiling of HLA-E/HLA-G with NKG2A/C-expressing NK cells, functional validation in EC organoid or *ex vivo* models, and incorporation of NK-cell pharmacodynamic endpoints into clinical trials. Importantly, the extent to which NK-cell dysfunction differs across EC molecular subtypes remains insufficiently defined, as current subtype-stratified studies more consistently demonstrate immune variation at the level of CD8^+^ T cells than NK cells.

To translate these biological observations into clinically actionable strategies, EC molecular and immune subtypes should be linked to dominant NK-evasion mechanisms, candidate NK-directed interventions, and measurable biomarkers or pharmacodynamic endpoints (**Table 1**).

**Table 1:** Endometrial carcinoma molecular subtypes, putative NK-cell evasion mechanisms, and candidate NK-directed interventions.

EC subtype	Immune phenotype	Putative NK-evasion mechanisms	Candidate NK-directed interventions	Biomarkers/endpoints to measure response	References
POLE-ultramutated	Immune-hot; very high TMB, abundant neoantigens, and prominent T-cell infiltration. NK-cell contribution remains incompletely defined.	Checkpoint-rich context; possible NKG2A, KIR, TIGIT, and TIM-3 engagement. NK-specific evasion remains insufficiently defined.	ICI is clinically relevant in selected settings; NK-checkpoint blockade or cytokine activation remain investigational and biomarker-driven.	POLE status; TMB; PD-1/PD-L1; NK-cell density; NKp46; CD56/CD16 subsets; NKG2A/NKG2C; TIGIT/TIM-3; spatial NK-tumor proximity.	(15, 16, 20–22, 31, 32)
MSI-H/dMMR	Immune-inflamed; high mutational burden, neoantigen load, and frequent checkpoint expression.	Checkpoint-rich, inflammatory but potentially suppressive TME; possible HLA-E/HLA-G-mediated NK inhibition and cytokine suppression in resistant disease.	PD-1 blockade is established in biomarker-defined settings; NK-directed approaches may be explored in ICI-resistant or relapsed disease within rational combinations.	MMR/MSI status; TMB; PD-1/PD-L1; HLA-E/HLA-G; NKG2A/NKG2C; TIGIT/TIM-3; NK cytotoxic gene signatures; longitudinal NK activation markers.	(23, 24, 26, 27, 31, 32, 105, 119, 137)
NSMP/copy-number low	Heterogeneous, often pMMR/MSS, and less inflamed than POLE-mutated or MSI-H/dMMR tumors.	Immune exclusion, stromal suppression, impaired NK recruitment, TGF-β/IL-6 signaling, lactate-rich metabolism, hypoxia/HIF-1α, EV communication, and possible NKG2D-ligand dysregulation.	Candidate options include NK-cell engagers, IL-15 activation, TGF-β modulation, metabolic/hypoxia-targeted TME modulation, adoptive NK approaches, and pembrolizumab-lenvatinib combinations where appropriate.	NK infiltration; CXCL12/CCL21/CCL27; TGF-β/IL-6; lactate/LDHA; HIF-1α; EV/exosomal miRNA cargoes; NKG2D ligands; soluble MICA/B; NK degranulation/cytotoxicity; pharmacodynamic NK activation.	(11, 18, 28, 29, 36, 38, 41, 43, 82, 92–104, 116, 121, 136, 138, 231)
p53-abnormal/copy-number high	Often immune-cold/excluded, with aggressive biology and low effector-cell infiltration; HER2 amplification may occur in selected tumors.	Low NK/T-cell infiltration, suppressive TME, possible HLA-E/HLA-G inhibition, and antigen heterogeneity. HLA-I loss may increase NK susceptibility, but local suppression may limit this effect.	Options include adoptive NK cells, CAR-NK platforms, NK-cell engagers, and TME-modulating combinations. Antigen-directed strategies require careful target selection and safety monitoring.	HER2 or other target-antigen expression; HLA-E/HLA-G; NK trafficking; NK persistence; cytotoxicity markers; cytokine release; on-target/off-tumor safety endpoints.	(15, 16, 30, 36, 38, 105, 119, 123, 136, 138, 139, 141, 170, 224–228, 232)
Recurrent/immunotherapy-resistant EC	Primary or acquired treatment resistance, often with suppressive or poorly inflamed immune contexture.	Checkpoint redundancy, suppressive cytokines, impaired NK recruitment/persistence, stromal barriers, lactate, hypoxia, tumor-derived EVs, and loss/shedding of activating ligands.	Combinations of ICIs, NK-checkpoint blockade, cytokines, NK-cell engagers, adoptive NK cells, CAR-NK approaches, and TME-modulating strategies should be guided by subtype, target expression, and pharmacodynamic monitoring.	Longitudinal NK phenotyping; spatial receptor–ligand profiling; circulating NK activation; lactate/LDHA; HIF-1α; circulating EV/exosomal cargoes; soluble MICA/B; cytokine release; persistence; response and resistance endpoints.	(11, 41, 43, 48, 92–105, 116, 121–123, 136–139, 170, 224–229, 231, 232)

ICI, immune checkpoint inhibitor; NK, natural killer; TMB, tumor mutational burden; TME, tumor microenvironment.

## 4 Immunotherapy approaches in endometrial cancer

Immunotherapy aims to restore or enhance anti-tumor immune responses through different strategies, including monoclonal antibodies, cytokines, effector-cell therapies, ICIs, vaccines, bispecific antibodies, and engineered immune cells (124–126). In EC, both innate and adaptive immunity can contribute to therapeutic responses. While T cells remain the best-characterized immune effectors, NK cells may complement adaptive immunity by directly recognizing stressed or HLA-I-altered tumor cells and by shaping DC and T-cell responses.

A subset of EC, particularly dMMR/MSI-H tumors, is highly immunogenic because of increased TMB and neoantigen formation, which can promote tumor-specific T-cell responses (127–133). By contrast, pMMR/MSS tumors are generally less responsive to ICI monotherapy, supporting the need for additional or combination approaches able to modulate the TME and recruit innate effector mechanisms (134). In this section, we first discuss NK-directed therapeutic strategies and then summarize ICI-based and other emerging immune-based approaches in EC, highlighting how these modalities may be integrated to improve responses in less immunogenic or treatment-resistant disease (127, 135) (**Tables 1**, **2**).

**Table 2:** Clinical studies involving immune-based strategies, NK-related approaches, oncolytic viruses, and engineered immune cells in endometrial cancer.

Therapeutic category	Study/trial	NCT number/status	EC setting	Intervention/target	Target rationale in EC and potential NK-related effects or constraints	Sponsor/study type
CAR-NK/engineered NK cells	CLDN6/GPC3/Mesothelin/AXL-CAR-NK Cell Therapy for Advanced Solid Tumors	NCT05410717; recruiting	Advanced solid tumors including recurrent EC	CAR-NK cells targeting CLDN6, GPC3, mesothelin, or AXL	Exploratory EC relevance depends on target expression. NK-specific constraints include tumor trafficking, persistence, antigen heterogeneity, and on-target/off-tumor toxicity.	Second Affiliated Hospital of Guangzhou Medical University; interventional
Cytokine/NK-receptor biology	Cytokine Regulation of Natural Killer Receptors in Inhibiting Activated T-Cell Function	NCT00173290; unknown status	Includes endometrial, cervical, breast, and other cancers	Observational analysis of cytokines and NK receptor biology	Relevant to NK-receptor regulation and cytokine-driven immune suppression/activation; not an EC-specific therapeutic trial.	National Taiwan University Hospital; observational
Metabolic/mTOR modulation	Metformin and Temsirolimus in Metastatic or Unresectable Solid Tumors or Lymphoma	NCT00659568; completed	Includes EC among advanced solid tumors	Metformin plus temsirolimus	May indirectly influence NK-cell activation, tumor infiltration, metabolic stress, and sensitivity to immune-mediated killing; NK effects are exploratory.	London Health Sciences Centre; interventional
Cytokine + antibody/chemotherapy	Interleukin-12, Paclitaxel, and Trastuzumab in Solid Tumors	NCT00028535; completed	Includes recurrent endometrial carcinoma	Recombinant IL-12, paclitaxel, trastuzumab	IL-12 can enhance NK-cell IFN-gamma production and ADCC in antibody-based therapy; applicability depends on HER2 expression and immune competence.	National Cancer Institute; interventional
Vaccine + ICI + cytokine agonist	Pembrolizumab, Lenvatinib and IL-15 Superagonist N-803 with HER2-Targeting Autologous Dendritic Cell Vaccine	NCT06253494; recruiting	Advanced or metastatic EC	Pembrolizumab, lenvatinib, N-803, AdHER2DC vaccine	N-803 may expand/activate NK and CD8+ T cells; HER2-directed vaccine rationale depends on HER2 expression. Monitor cytokine toxicity, NK activation, and TME remodeling.	National Cancer Institute; interventional
TIL assessment	Tumor-Infiltrating Lymphocytes in Endometrial Cancer	NCT06976333; completed	Endometrial cancer tissue study	Diagnostic assessment of TILs and imaging-derived features	Primarily T-cell/TIL focused; can contextualize immune infiltration but does not directly test NK-cell function.	Jagiellonian University; observational
Adoptive TIL therapy	Lifileucel in Adults with Advanced Endometrial Cancer	NCT06481592; recruiting	Advanced EC	Lifileucel/TIL therapy	T-cell focused adoptive therapy; relevant comparator for NK-cell strategies and for immune-infiltrated EC phenotypes.	Iovance Biotherapeutics; interventional
Adoptive TIL therapy	DeTIL-0255 in Adults with Advanced Malignancies	NCT05107739; terminated	Advanced malignancies including EC	DeTIL-0255 drug product	TIL-based approach; useful background for cellular therapy feasibility in gynecologic cancers.	Nurix Therapeutics; interventional
Oncolytic virus	Oncolytic Virus Injection R130 for Relapsed/Refractory Cervical and Endometrial Cancer	NCT05812677; recruiting	Relapsed/refractory cervical and EC	Recombinant oncolytic HSV-1 R130	Oncolytic viruses may induce immunogenic tumor-cell death, enhance antigen release, and promote local innate immune activation, including NK-cell recruitment/function.	Shanghai Yunying Medical Technology; interventional
Oncolytic virus	H101 with or without Radiotherapy in Refractory/Recurrent Gynecological Malignancies	NCT05051696; active, not recruiting	Refractory/recurrent female genital neoplasms	Intratumoral H101 with or without radiotherapy	Potential to remodel local inflammation and enhance innate and adaptive anti-tumor responses; NK endpoints should be added where feasible.	Liu Zi; interventional
Oncolytic virus + JAK inhibition	VSV-hIFNbeta-NIS with or without Ruxolitinib Phosphate	NCT03120624; active, not recruiting; with results	Stage IV or recurrent EC	VSV-hIFNbeta-NIS with or without ruxolitinib	Viral oncolysis and type I IFN may activate innate immunity; ruxolitinib may modulate cytokine signaling and requires careful immune-monitoring.	Mayo Clinic; interventional
ICI in molecularly selected EC	Pembrolizumab in Ultramutated and Hypermutated Endometrial Cancer	NCT02899793; completed; with results	Recurrent POLE-ultramutated or hypermutated EC	Pembrolizumab	Primarily T-cell checkpoint therapy; NK-related endpoints could include PD-1^+^ NK cells, cytotoxic gene signatures, and spatial immune infiltration.	Yale University; interventional
CAR-T/antigen-targeted cellular therapy	CART-meso for Relapsed and/or Chemotherapy-Refractory Advanced Malignancies	NCT02580747; unknown status	Mesothelin-positive tumors including EC	Anti-mesothelin CAR T cells	Target rationale depends on mesothelin expression. Constraints include trafficking, persistence, antigen heterogeneity, and on-target/off-tumor toxicity; informative for future CAR-^NK^ design.	Chinese PLA General Hospital; interventional
Antibody–drug conjugate/targeted immune therapy	CTIM-76 in Recurring Ovarian Cancer and Other Advanced Solid Tumors	NCT06515613; recruiting	Includes EC	CTIM-76	Target rationale in EC should be defined by antigen expression. Potential NK relevance is indirect unless Fc-mediated ADCC or immune remodeling is documented.	Context Therapeutics; interventional
Bispecific/immune checkpoint-targeted antibody	Ivonescimab in Endometrial and Cervical Cancers	NCT06925724; recruiting	Endometrial and cervical cancers	Ivonescimab	Dual PD-1/VEGF targeting may modulate checkpoint inhibition and angiogenesis; potential indirect benefit for NK trafficking and TME normalization.	Memorial Sloan Kettering Cancer Center; interventional
Bispecific antibody	GEN1047 for Solid Tumors	NCT05180474; active, not recruiting	Includes EC and ovarian cancer	GEN1047, B7-H4-directed bispecific antibody	B7-H4 expression may support EC target rationale. Primarily T-cell mediated; NK contribution may be indirect and should not be overstated.	Genmab; interventional
Bispecific antibody	XmAb541 in Advanced Solid Tumors	NCT06276491; recruiting	Includes EC	XmAb541	Target rationale and NK involvement depend on mechanism/Fc design; key constraints include antigen expression, safety, and immune-cell engagement profile.	Xencor; interventional
Bispecific/immunomodulatory antibody combination	REGN5668 with Cemiplimab, Cemiplimab + Fianlimab, or Ubamatamab	NCT04590326; recruiting	Gynecologic cancers including EC-related cohorts	REGN5668 combinations	Combination checkpoint/bispecific strategies may reshape immune engagement. NK-related endpoints should include Fc-mediated activity, NK activation, and cytokine release.	Regeneron; interventional
Bispecific checkpoint antibody	Vudalimab/XmAb20717 in Selected Advanced Gynecologic and Genitourinary Malignancies	NCT05032040; active, not recruiting; NCT03517488; completed	Includes EC among selected tumors	Vudalimab/XmAb20717	Dual checkpoint targeting may indirectly affect NK-cell function if PD-1^+^ NK subsets are present; main expected mechanism is T-cell reinvigoration.	Xencor; interventional
Targeted antibody/combination therapy	SSGJ-707 in Advanced Gynecologic Cancer	NCT06522828; recruiting	Advanced/recurrent EC and platinum-resistant ovarian cancer	SSGJ-707 with or without chemotherapy components	Potential immune relevance depends on target and Fc engineering; monitor immune activation, ADCC potential, and standard safety endpoints.	Sunshine Guojian Pharmaceutical; interventional
MUC16-targeted bispecific antibody	Ubamatamab Alone or with Cemiplimab in Recurrent MUC16+ Cancers	NCT03564340; recruiting	Recurrent MUC16+ cancers including EC-related cohorts if MUC16-positive	Ubamatamab, cemiplimab, sarilumab combinations	Target rationale depends on MUC16 expression. Cytokine release, antigen heterogeneity, and immune-cell engagement should be monitored.	Regeneron; interventional

ADCC, antibody-dependent cellular cytotoxicity; CAR, chimeric antigen receptor; EC, endometrial cancer; ICI, immune checkpoint inhibitor; IFN, interferon; NK, natural killer; TIL, tumor-infiltrating lymphocyte; TME, tumor microenvironment; VSV, vesicular stomatitis virus.

### 4.1 NK cells as therapeutic effectors against EC

**Figure 2:**
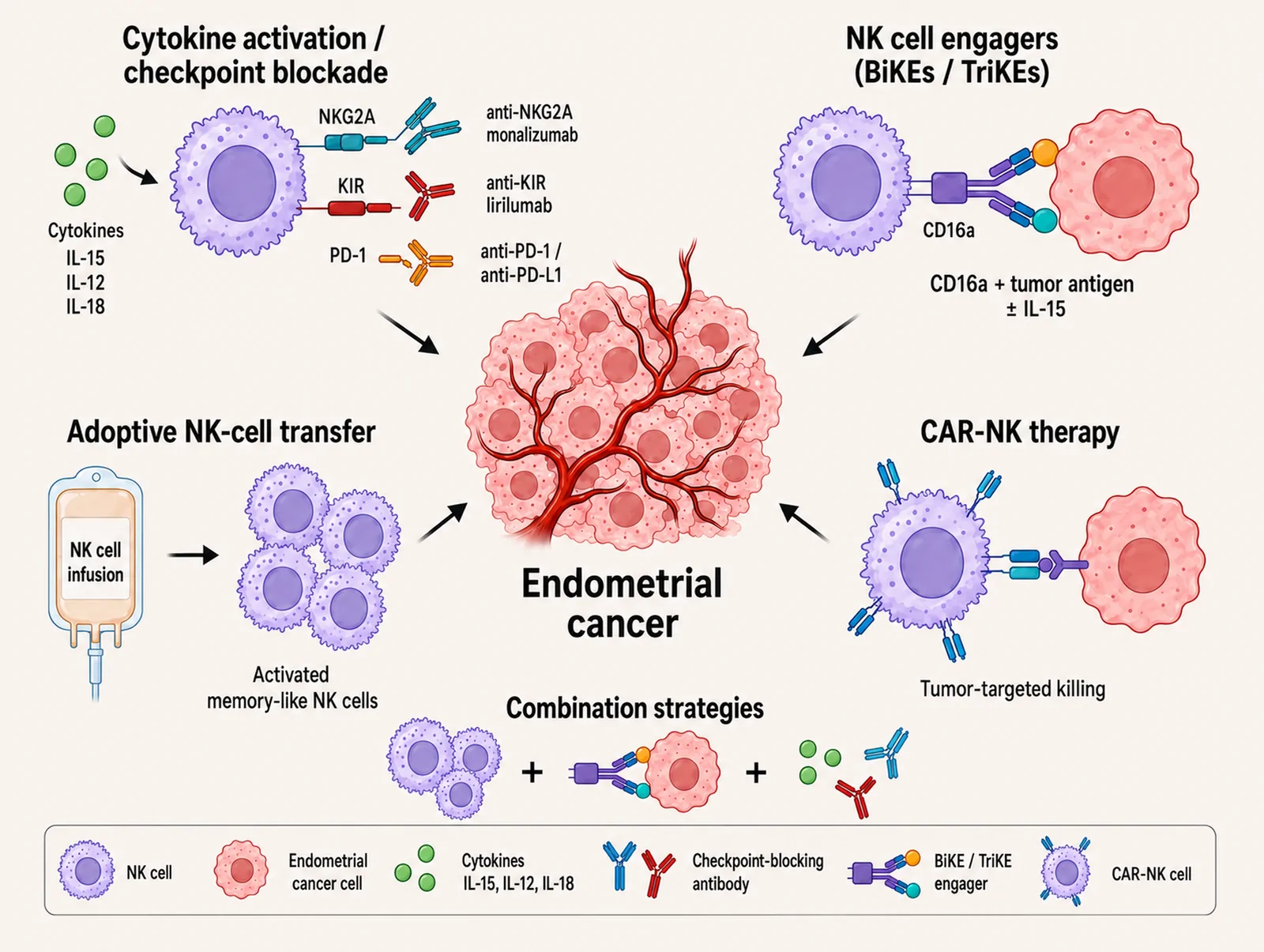
NK-cell-based therapeutic strategies in EC. Schematic overview of therapeutic approaches aimed at restoring or enhancing natural killer (NK) cell-mediated anti-tumor immunity in endometrial cancer. Cytokine-based stimulation with IL-15, IL-12, and IL-18 may promote NK-cell activation, survival, and cytotoxic effector function. Blockade of inhibitory pathways, including NKG2A, KIR, and PD-1/PD-L1, may counteract NK-cell suppression within the tumor microenvironment. NK-cell engagers, including bispecific and trispecific killer engagers (BiKEs/TriKEs), are designed to redirect NK cells toward tumor-associated antigens through CD16a engagement, with selected TriKE platforms incorporating IL-15 to enhance NK-cell persistence and function. Adoptive transfer of activated or memory-like NK cells may increase the pool of cytotoxic effector cells, whereas CAR-NK-cell therapy enables antigen-directed tumor recognition and killing. These strategies may be applied alone or in rational combinations to overcome NK-cell dysfunction and reinforce anti-tumor immunity in EC.

Based on NK-cell biology and dysfunction in EC, strategies that enhance NK-cell number, activation, trafficking, persistence, or tumor recognition may have therapeutic relevance (**Figure 2**) (136). **Table 1** summarizes candidate NK-directed approaches according to EC molecular subtype and immune phenotype. Among these approaches, NK-cell engagers, including bispecific and trispecific killer engagers (BiKEs/TriKEs), represent candidate strategies to improve tumor recognition by innate effector cells (136).

Because many current immunotherapy trials in EC are not NK-directed, we distinguish NK-centered strategies from approaches with indirect relevance to innate immunity. Standard ICI trials, TIL-based approaches, and oncolytic-virus studies are therefore discussed mainly as contextual frameworks for understanding resistance mechanisms and rational NK-complementary combinations.

Recent evidence suggests that NK-cell biology may be clinically relevant in specific EC subgroups. In dMMR EC, activated CD16^+^ NK-cell signatures were associated with immune checkpoint blockade (ICB)-responsive epigenetic dMMR tumors, whereas mutational dMMR tumors appeared more dependent on CD8^+^ T-cell responses (137). In a study combining single-cell RNA sequencing of EC cells and matched T-cell receptor sequencing of peripheral blood mononuclear cells (PBMCs) from 24 EC patients, only a subset of patients with epigenetic dMMR responded to anti-PD-1 antibody pembrolizumab. Notably, CD16^+^ NK cells were significantly associated with OS, and a four-gene NK-related signature, termed epiRNK4 and including CD63, PPIB, CEBPB, and LDOC1, was associated with survival in multivariate analysis (137). These data suggest that NK-cell states may contribute to response heterogeneity within dMMR EC.

CAR-NK cells represent a promising strategy for gynecological cancers, alongside CAR-T-cell approaches (138–143). The main challenge is to identify tumor-associated or TME-associated targets that are sufficiently expressed in EC while limiting on-target/off-tumor toxicity. In addition, engineered NK cells must traffic efficiently into tumor tissue, persist long enough to exert cytotoxicity, and maintain function within an immunosuppressive TME (41–43). In EC, candidate targets may be identified through proteogenomic analyses and phenotypic characterization of patient-derived organoids (144–153). Potential antigenic targets for CAR-based strategies in EC include mesothelin (MSLN), human epidermal growth factor receptor 2 (HER2), Müllerian inhibiting substance type II receptor (MISIIR), folate receptor alpha (FRα), and alkaline phosphatase placental type (ALPP) (154–160). These antigens are overexpressed in selected EC contexts compared with healthy endometrium, supporting their investigation for antigen-directed cellular therapy. The tolerability and early clinical experience of selected HER2- or ALPP-directed CAR approaches suggest that NK-cell features, including innate cytotoxicity and recognition of stress ligands such as UL16-binding protein (ULBP) and MICA/B proteins, may be leveraged to improve antigen-directed killing in EC (161, 162). CAR-NK cells may kill tumor targets through both CAR-dependent activation and intrinsic NK cytotoxic programs (161, 162). This property has encouraged the design of CAR constructs with calibrated activation capacity, including constructs with reduced costimulatory strength or non-activating/adhesion-oriented modules intended to improve tumor localization while limiting excessive off-tumor activation (163, 164). Multi-target CAR-NK approaches may also help reduce immune escape driven by loss or downregulation of a single target antigen (165).

CAR-NK constructs should account for the relatively short lifespan of NK cells compared with T cells (166). Incorporation of cytokine-support modules, especially IL-15, is one strategy to enhance NK-cell activation, proliferation, survival, and clinical efficacy (138). IL-15 can be delivered as a soluble or membrane-bound module in armored CAR-NK cells; however, soluble IL-15 may increase systemic inflammatory toxicity, whereas membrane-bound IL-15 has been proposed to improve antitumor activity while limiting undesired inflammatory effects (138). Additional armored-CAR strategies include gene-editing approaches aimed at increasing tumor-cell susceptibility to apoptosis or protecting NK cells from pro-apoptotic programs, thereby prolonging CAR-NK persistence and increasing killing efficiency (167–169).

Another key point in CAR-NK therapy is the possibility of using allogeneic NK cells rather than patient-specific autologous CAR-T cells (170, 171). Innate and innate-like immune-cell platforms, including NK cells, monocytes, NKT cells, and γδ T cells, generally do not recognize tissue MHC in the same way as conventional T cells, supporting the rationale for off-the-shelf manufacturing from allogeneic sources (170, 171). For CAR-NK products, potential sources include peripheral blood, umbilical cord blood, embryonic stem-cell precursors, and induced pluripotent stem cells (iPSCs), although expansion protocols remain complex and costly (172). Feeder-cell-based expansion may improve NK-cell yield but can introduce manufacturing and safety concerns, including the theoretical risk of contaminating feeder cells triggering alloreactivity (170). The NK-92 cell line is readily expandable and engineerable, but its malignant origin requires irradiation of the final cellular product, thereby limiting *in vivo* persistence (173). Donor selection remains an important consideration, and NKG2C^+^ adaptive-like NK-cell enrichment together with favorable HLA/KIR mismatch may enhance activating signals while reducing inhibitory KIR-mediated signaling (170, 174, 175). CAR-NK cells may also undergo trogocytosis, acquiring tumor target antigens and thereby promoting fratricide or loss of function; inhibitory KIR-CAR systems have been proposed to limit trogocytosis-associated CAR-NK cell death and exhaustion (176).

Several NK-cell-specific strategies for CAR-NK engineering are also being developed. Because NK cells express multiple activating receptors, including DNAM-1, NKp46, and NKG2D, CAR designs can incorporate NK-adapted signaling modules, such as NKp46-DNAM-1/CD3ζ pairs or NKp46 fused to CD3ζ, DAP10, or DAP12 (177–179). These constructs may better exploit endogenous NK-cell signaling architecture than CAR designs directly adapted from T cells, although they have not yet been validated in EC. Another important limitation is poor infiltration and persistence within solid tumors, where fibroblasts, TGF-β, myeloid cells, adenosine, hypoxia, and other suppressive factors can downregulate NK-cell activity (180, 181). In this context, CAR-based strategies may target either tumor cells or TME components. For example, anti-fibroblast activation protein (FAP) CAR-NK cells showed activity in cervical cancer models using NK-92 or cord-blood-derived NK cells engineered with a third-generation anti-FAP CAR (140). This may be relevant to EC because tumor-associated fibroblasts can promote EC progression through the stromal cell-derived factor 1/C-X-C motif chemokine ligand 12 (SDF-1/CXCL12)–CXCR4 axis (182). In addition, anti-FAP CAR-T cells showed minimal CAR-related toxicity in a phase I trial in malignant pleural mesothelioma, supporting the feasibility of targeting FAP-positive stromal elements in selected settings (183). Although direct EC-specific CAR-NK evidence remains limited, ongoing CAR-T-cell studies in EC targeting mesothelin, ROR1, and HER2 may help define feasibility, safety, and target rationale for future CAR-NK design (**Table 2**).

Innate and innate-like platforms also include CAR-engineered invariant natural killer T (CAR-iNKT) cells (171). In particular, allogeneic mesothelin-directed CAR-NKT cells generated from hematopoietic stem and progenitor cells have been proposed as a potentially scalable strategy able to target EC cells and immunosuppressive CD1d^+^ myeloid populations (171). Mesothelin-targeting memory-like CAR-NK cells armored with IL-12 have also been reported to increase metabolic fitness, cytotoxicity, and IFN-γ production compared with non-armored CAR-NK cells (184). Overall, CAR-NK, CAR-NKT, and CAR-monocyte/macrophage strategies may provide complementary mechanisms of action, including antigen-directed recognition, intrinsic NK cytotoxicity, cytokine production, phagocytosis, antigen presentation, and secondary T-cell activation (170, 171, 184, 185). Their development in EC should incorporate key barriers such as trafficking, persistence, antigen heterogeneity, immunosuppressive cytokines, and on-target/off-tumor toxicity into trial design and biomarker monitoring.

### 4.2 EC treatment with immune checkpoint inhibitors

ICI therapy in EC is primarily supported by restoration of anti-tumor T-cell responses through blockade of inhibitory receptor–ligand interactions (186). However, checkpoint blockade may also influence innate immunity indirectly through changes in cytokine signaling, angiogenesis, antigen release, and PD-L1-dependent crosstalk with NK cells. Therefore, ICI-based trials are discussed here only insofar as they define the clinical resistance context in which NK-directed or NK-complementary strategies may be integrated (186–195).

Clinically, ICI therapy is most relevant in recurrent, advanced, or metastatic EC (186, 192, 196). Pembrolizumab demonstrated activity in advanced dMMR/MSI-H EC, and dostarlimab showed sustained responses in the GARNET trial, supporting PD-1 blockade in biomarker-defined dMMR/MSI-H disease after platinum-based therapy (12, 196, 197). Because regulatory indications are rapidly evolving, the precise approved setting, line of therapy, and biomarker-defined subgroup should be verified at the time of submission.

Combination ICI-based regimens have further reshaped the therapeutic landscape. In KEYNOTE-775, lenvatinib plus pembrolizumab improved progression-free survival (PFS) and OS compared with chemotherapy in patients with advanced EC, including both pMMR and dMMR disease (11). However, toxicity was substantial, with grade ≥3 adverse events occurring more frequently in the lenvatinib–pembrolizumab arm (11). Conversely, the phase III LEAP-001 trial evaluating first-line lenvatinib plus pembrolizumab versus chemotherapy in advanced EC did not improve PFS or OS in pMMR disease, although toxicity was manageable (198). In the NRG-GY018 trial, pembrolizumab plus carboplatin–paclitaxel significantly improved PFS compared with chemotherapy alone, independently of mismatch repair status, whereas OS data were still immature at the time of reporting (131).

Real-world treatment patterns also highlight the need for additional therapeutic options. In a retrospective analysis of patients with primary advanced or recurrent EC in the United States, recommended first-line treatment was not applied in approximately 40% of cases across a large multi-site cohort (199). Treatment regimens varied widely and included platinum-based chemotherapy, non-platinum chemotherapy, VEGF-targeted therapy, HER2-directed therapy, hormonal therapy, mTOR-based therapy, PD-1/PD-L1 monotherapy or combinations, and other approaches (199). This heterogeneity likely reflects patient-specific factors, evolving evidence, and the need for more effective first-line and later-line strategies, including immunotherapies and NK-complementary approaches.

Hypothetically, ICI therapy could trigger NK-cell-mediated immune responses or limit inhibition of PD-1^+^ CAR-NK cells, depending on PD-L1 expression on EC cells. The expression and functional relevance of PD-1 and cytotoxic T-lymphocyte-associated protein 4 (CTLA-4) on NK cells remain controversial (200, 201). Nevertheless, selected preclinical and translational studies indicate that PD-1/PD-L1 blockade can affect NK-cell function and may contribute to the efficacy of ICB in some models (200–202). In ovarian carcinoma, PD-1^+^ NK cells have been reported in ascites, where PD-1 expression was associated with impaired degranulation that could be reversed by anti-PD-L1 antibodies (202). Thus, it is plausible that ICI treatment may influence the behavior of tumor-infiltrating NK cells. In addition, CAR-NK cells engineered to target PD-L1 have shown enhanced *in vitro* and *in vivo* antitumor activity, and combination with nivolumab produced synergistic antitumor effects in humanized mouse models (203). These findings suggest that CAR-NK approaches targeting PD-L1-expressing tumors, including selected EC contexts, warrant further preclinical evaluation before clinical translation.

### 4.3 Novel and alternative therapeutic approaches for advanced EC

Despite recent progress, recurrent or metastatic EC remains difficult to treat, particularly after first-line therapy. This supports the development of new combinations and alternative immune-based approaches. One rational strategy is to identify tumor-associated antigens, neoantigens, or gene fusion products across EC subgroups and exploit them for vaccine or cellular immunotherapy development (204–209). The tissue-agnostic approval of pembrolizumab for MSI-H/dMMR tumors further supports the principle of biomarker-defined immunotherapy across tumor types (210).

Proteogenomic characterization of EC by the Clinical Proteomic Tumor Analysis Consortium integrated DNA, RNA, protein, phosphorylation, and acetylation data from 95 tumors, including 83 endometrioid and 12 serous tumors (208). This analysis confirmed the relevance of p53 and WNT/β-catenin pathways and identified extensive post-translational regulation. Tumors were classified according to TCGA molecular groups, including POLE, MSI, copy number variation (CNV)-low, and CNV-high subtypes (15, 208). Approximately half of the tumors expressed putative neoantigen proteins, with the highest neoantigen burden in POLE-mutated tumors. Cancer/testis antigens, including IGF2BP3, ATAD2, and PBK, were detected independently of POLE or MSI status, suggesting that antigen discovery should not be restricted to hypermutated tumors (208).

Additional biomarkers may refine patient selection for immunotherapy (210). POLE-hotspot tumors show immunogenic features and neoantigen profiles that may help define immunotherapy-sensitive subsets beyond MSI status (209). Epigenetic mechanisms may also contribute to immune suppression, as DNA methylation has been linked to an immunosuppressive microenvironment in metastatic endometrial clear cell carcinoma (211). Multi-omics analyses have identified candidate EC antigens, including PGR, RBPJ, PARVG, and MSX1, and described immune subtypes with different TMB, MSI, immune, stromal, and CNV features (204). Additional immune profiling studies have highlighted distinct immune microenvironmental features across EC molecular subtypes (37). Two immune subtypes, C1 and C2, have also been described, with the C2 subtype showing higher TMB, MSI, immune and stromal scores, and lower CNV burden, consistent with an immune-hot phenotype potentially more susceptible to immunotherapy (37).

The following TIL and oncolytic-virus approaches are not NK-directed therapies. They are retained as contextual examples of immune-based strategies that may remodel antigen release, inflammatory tone, and effector-cell recruitment, and may therefore inform future NK-complementary combinations.

Tumor-infiltrating lymphocytes may also represent a cellular adoptive therapy platform in EC (212–217). Manufacturing feasibility has been evaluated in a small cohort of 11 EC patients using Iovance’s 22-day Gen2 process, with successful TIL expansion in 10 of 11 cases and variable IFN-γ responses among tested samples (212). These findings provided the rationale for the phase II IOV-END-201 trial (212). However, the feasibility, yield, viability, clonality, immune phenotype, and anti-tumor cytotoxicity of EC-derived TILs require further validation (212).

Oncolytic viruses may also enhance anti-tumor immunity in EC (218–220). These agents preferentially infect and lyse tumor cells, promoting antigen release and immune activation. Preclinical evidence supports the activity of vaccinia virus, measles virus, and vesicular stomatitis virus in EC models (218–220). Clinical evaluation is ongoing for R130, a modified HSV-1 encoding anti-CD3 scFv, CD86, PD-1-related immunomodulatory elements, and HSV2-US11, administered intraperitoneally or intratumorally in trial NCT05812677. Primary endpoints include adverse events and systemic immune response, whereas secondary endpoints include disease assessment, disease control rate, and quality of life (221, 222). Results are expected in 2026. Similar oncolytic virus approaches are also being explored across several tumor types, including gynecologic cancers (**Table 2**) (222, 223).

#### 4.3.1 NK-cell platforms as stimulators of anti-tumor adaptive immunity

The identification of specific antigens in EC supports the development of T-cell-directed therapeutic strategies. However, NK cells may offer complementary advantages, particularly through the generation of allogeneic CAR-NK platforms derived from NK-cell lines, such as NK-92 cells (173, 175, 224), or from iPSCs (225–228). Compared with peripheral blood- or umbilical cord blood-derived NK cells, NK-92- and iPSC-derived NK cells may offer advantages in terms of standardized manufacturing, scalability, and genetic engineering (228).

NK cells recognize self-MHC molecules through several inhibitory receptors, including leukocyte immunoglobulin-like receptors (LILRs), killer-cell immunoglobulin-like receptors (KIRs), and NKG2 family members (229). These receptors deliver inhibitory signals; therefore, reduced expression of selected inhibitory receptors may facilitate stronger NK-cell-mediated anti-tumor responses. NK-92 cells and iPSC-derived NK cells usually express few, if any, of these receptors, supporting their use as homogeneous allogeneic platforms for CAR engineering. CAR-NK cells may therefore combine CAR-mediated antigen recognition with the intrinsic cytolytic activity of NK cells.

A recent iPSC-derived CAR-NK platform provides proof of concept for this approach in solid tumors (227). In this system, iPSCs were engineered to express multiple functional modules, including CCL19, CCR2B, high-affinity CD16, IL-15, and the NKG2D–DAP10 complex. NKG2D and CD16 enhanced anti-tumor activity, IL-15 supported NK-cell persistence, CCR2B improved tumor localization, and CCL19 promoted DC recruitment (227). This strategy illustrates how next-generation CAR-NK platforms may address major limitations of cellular therapy in solid tumors, including trafficking, persistence, sustained cytotoxicity, and integration with adaptive immunity.

Bispecific monoclonal antibodies may also contribute to anti-tumor immunity by redirecting immune effector cells and modulating the local TME. Although many bispecific formats primarily engage T cells, localized immune activation and cytokine release may indirectly enhance NK-cell activity by reducing immunosuppression and increasing tumor-cell stress signals. Conversely, CAR-NK cells or activated NK cells may promote antigen release and DC activation, thereby facilitating subsequent T-cell responses. Overall, rational combinations of CAR-NK cells, bispecific antibodies, ICIs, cytokines, and other immunotherapeutic tools may be required to overcome the limitations of single-agent approaches in advanced or resistant EC (230).

## 5 Conclusions and future perspectives

NK-cell dysfunction is emerging as a relevant component of immune escape in EC. Although NK-cell infiltration, phenotype, and function remain less extensively characterized than T-cell responses, available data support a potential contribution of NK cells to disease progression, immunotherapy resistance, and biomarker development. These observations support the integration of NK-cell parameters into future immune-monitoring strategies, particularly in molecular subgroups with limited T-cell-mediated responsiveness.

The emerging understanding of NK-cell biology in EC highlights several mechanisms of dysfunction, including altered activating and inhibitory receptor signaling, immune checkpoint engagement, impaired chemokine-mediated recruitment, and suppression by stromal and cytokine-mediated networks within the TME. Targeting these pathways may provide a rational strategy to restore NK-cell cytotoxicity and reinforce anti-tumor immune surveillance.

Therapeutic approaches aimed at reactivating endogenous NK cells or augmenting NK-cell activity through *ex vivo* expanded NK cells, cytokine-induced memory-like NK cells, NK-cell engagers, and CAR-NK platforms hold considerable promise. Their integration with current treatment modalities, including chemotherapy, radiotherapy, hormonal therapy, targeted therapy, and ICB, may broaden therapeutic opportunities, especially for patients with recurrent, immune-cold, or immunotherapy-resistant EC.

Future studies should translate these observations into a structured biomarker-development roadmap. NK-cell-related parameters should be analyzed together with established T-cell immune markers and integrated into current EC molecular classification frameworks, including POLE-mutated, MSI-H/dMMR, NSMP/pMMR-MSS, and p53-abnormal tumors. In this setting, combined T-cell and NK-cell gene signatures may provide more informative immune profiles than T-cell-centered approaches alone and may help refine immunotherapy response prediction models. This will require spatial assessment of NK-cell localization in relation to HLA-E, HLA-G, NKG2A, NKG2C, and activating ligands; receptor–ligand profiling of inhibitory and activating NK-cell axes; evaluation of cytotoxic gene signatures and circulating NK-cell phenotypes; and paired tumor–blood analyses to distinguish local NK-cell dysfunction from systemic immune alterations. Functional validation in EC organoid or *ex vivo* models, together with longitudinal pharmacodynamic monitoring of NK-cell activation in clinical trials, will be particularly important for pMMR/MSS and immune-cold tumors, where NK-directed or NK-complementary strategies may help identify patients most likely to benefit from future combination therapies. Overall, NK-cell biomarkers should complement current molecular classification and support the development of biomarker-driven NK-directed immunotherapies in EC.

## Glossary

ADCC: antibody-dependent cellular cytotoxicity
ALPP: alkaline phosphatase placental type
APC: antigen-presenting cell
APM: antigen-processing and presentation machinery
BiKE: bispecific killer engager
CAR: chimeric antigen receptor
CAR-NK: chimeric antigen receptor natural killer cell
CAR-NKT: chimeric antigen receptor natural killer T cell
CAR-T: chimeric antigen receptor T cell
CNV: copy number variation
CTLA-4: cytotoxic T-lymphocyte-associated protein 4
DC: dendritic cell
dMMR: mismatch repair-deficient
dNK: decidual NK
DNAM-1: DNAX accessory molecule-1
EC: endometrial cancer
EMT: epithelial–mesenchymal transition
eNK: endometrial/uterine NK
ESGO/ESTRO/ESP: European Society of Gynaecological Oncology/European Society for Radiotherapy and Oncology/European Society of Pathology
EV: extracellular vesicle
FAP: fibroblast activation protein
FASL: Fas ligand
FIGO: International Federation of Gynecology and Obstetrics
FRα: folate receptor alpha
GM-CSF: granulocyte–macrophage colony-stimulating factor
HER2: human epidermal growth factor receptor 2
HIF-1α: hypoxia-inducible factor 1-alpha
HLA: human leukocyte antigen
HLA-E: human leukocyte antigen E
HLA-G: human leukocyte antigen G
HSC: hematopoietic stem cell
HSV-1: herpes simplex virus-1
ICB: immune checkpoint blockade
ICI: immune checkpoint inhibitor
IFN-γ: interferon gamma
ILC: innate lymphoid cell
ILT: immunoglobulin-like transcript
iPSC: induced pluripotent stem cell
KIR: killer cell immunoglobulin-like receptor
LDHA: lactate dehydrogenase A
LIF: leukemia inhibitory factor
LILR: leukocyte immunoglobulin-like receptor
MDSC: myeloid-derived suppressor cell
MHC: major histocompatibility complex
MICA/B: MHC class I chain-related A/B
MISIIR: Müllerian inhibiting substance type II receptor
MMR: mismatch repair
MSI: microsatellite instability
MSI-H: microsatellite instability-high
MSLN: mesothelin
mTOR: mechanistic target of rapamycin
NCR: natural cytotoxicity receptor
NKG2A: natural killer group 2A
NKG2C: natural killer group 2C
NKG2D: natural killer group 2D
NKT: natural killer T cell
NLRP3: nucleotide-binding oligomerization domain-like receptor protein 3
NK: natural killer
NSCLC: non-small cell lung cancer
NSMP: no specific molecular profile
ORR: objective response rate
OS: overall survival
PARP: poly-ADP-ribose polymerase
PBMC: peripheral blood mononuclear cell
PD-1: programmed cell death protein 1
PD-L1: programmed death-ligand 1
PDO: patient-derived organoid
PFS: progression-free survival
PlGF: placental growth factor
pMMR/MSS: mismatch repair-proficient/microsatellite-stable
SDF-1: stromal cell-derived factor 1/C-X-C motif chemokine ligand 12
TAF: tumor-associated fibroblast
TAM: tumor-associated macrophage
TCGA: The Cancer Genome Atlas
TGF-β: transforming growth factor beta
TIGIT: T-cell immunoreceptor with immunoglobulin and ITIM domain
TIL: tumor-infiltrating lymphocyte
TIM-3: T-cell immunoglobulin and mucin domain-containing protein 3
TIMP: tissue inhibitor of metalloproteinase
TMB: tumor mutational burden
TME: tumor microenvironment
TNF-α: tumor necrosis factor alpha
TRAIL: TNF-related apoptosis-inducing ligand
Treg: regulatory T cell
TriKE: trispecific killer engager
ULBP: UL16-binding protein
VEGF: vascular endothelial growth factor
VSV: vesicular stomatitis virus
